# Role of China in the Quest to Define and Control Severe Acute Respiratory Syndrome

**DOI:** 10.3201/eid0909.030390

**Published:** 2003-09

**Authors:** Robert F. Breiman, Meirion R. Evans, Wolfgang Preiser, James Maguire, Alan Schnur, Henk Bekedam, John S. MacKenzie

**Affiliations:** * International Centre for Diarrheal Disease Research, Bangladesh – Centre for Health and Population Research, Dhaka, Bangladesh; † National Public Health Service for Wales, Cardiff, United Kingdom; ‡ Institute for Medical Virology, Frankfurt, Germany; § Centers for Disease Control and Prevention, Atlanta, Georgia, USA; ¶ World Health Organization, Beijing, China; # University of Queensland, Brisbane, Australia*

**Keywords:** SARS, Severe acute respiratory syndrome, China, wildlife, animals, genetic, sequence, incidence, Seroprevalence, transmission, super-spreader, evolution, emergence, outcome

## Abstract

China holds the key to solving many questions crucial to global control of severe acute respiratory syndrome (SARS). The disease appears to have originated in Guangdong Province, and the causative agent, SARS coronavirus, is likely to have originated from an animal host, perhaps sold in public markets. Epidemiologic findings, integral to defining an animal-human linkage, may then be confirmed by laboratory studies; once animal host(s) are confirmed, interventions may be needed to prevent further animal-to-human transmission. Community seroprevalence studies may help determine the basis for the decline in disease incidence in Guangdong Province after February 2002. China will also be able to contribute key data about how the causative agent is transmitted and how it is evolving, as well as identifying pivotal factors influencing disease outcome. There must be support for systematically addressing these fundamental questions in China and rapidly disseminating results.

Severe acute respiratory syndrome (SARS) is a newly emerged disease, caused by a previously unknown coronavirus. The first known cases occurred in Guangdong Province in southern China in November and December 2002. During late February 2003, a physician who was incubating SARS traveled from Guangzhou, the provincial capital, to Hong Kong, Special Administrative Region of China, and stayed at a hotel. There, the virus was transmitted from him to local residents and to travelers, who became ill and transmitted disease to others when they returned to Vietnam, Singapore, Canada, and Taiwan, Province of China (*1*). SARS has now occurred in >8,450 people with >800 deaths worldwide.

The tally of SARS climbed rapidly in China through May 2003, then decelerated markedly during June. The disease has now been reported in 24 of China’s 31 provinces. By June 26, 2003, a total of 5,327 SARS cases and 348 deaths had been reported from mainland China, including 2,521 cases in Beijing and 1,512 in Guangdong Province.

Since February 2003, teams of technical consultants for the World Health Organization have been working in China to provide assistance to the Ministry of Health and provincial governments on public health responses to the SARS outbreak. A team that began working in China in March reviewed considerable clinical, epidemiologic, and laboratory data with scientists and officials from a variety of settings in Guangdong Province and Beijing. The team worked closely with colleagues from the National and Guangdong Provincial Centers for Disease Control, and together were able to establish that cases occurring in Guangdong beginning in November were clinically and epidemiologically similar to subsequent cases of SARS documented elsewhere.

The team observed detailed, comprehensive data collection forms, which are completed for activities and behaviors and clinical manifestations of patients with SARS. The team was informed that serum and respiratory secretion specimens collected from many patients from Guangdong were being held under appropriate storage conditions, awaiting further laboratory testing.

While a dedicated, collaborative international effort has resulted in substantial understanding of this disease with remarkable speed, critical information is still lacking. We detail a variety of knowledge gaps that should be addressed through a set of activities to optimize prevention and control of SARS within China and globally.

## Emergence of SARS-Associated Coronavirus in Humans

Available evidence suggests that SARS emerged in Guangdong Province, in southern China. How and when did it emerge? Did the causative agent evolve in an animal species and jump to humans (or perhaps first to other animal species), or did the virus evolve within humans? The genetic sequence of the virus has been obtained in several laboratories, and phylogenetic analyses have shown that it is unlike other coronaviruses of animal and human origin. Indeed, the virus has been tentatively placed in a new fourth genetic group (*2*,*3*).

Why is it so important to answer the question of how SARS emerged? Most recently recognized novel emergent viruses have been zoonotic, usually with a reservoir in wildlife (*4*,*5*). Thus, SARS coronavirus, if zoonotic, may provide the basis for modeling and predicting the appearance of other potential zoonotic human pathogens. More importantly, the information may be crucial for control of SARS. If this disease is to be curtailed or eliminated by strict public health measures, blocking further animal-to-human transmission will be critical. Only about half of the cases in Guangdong are attributed to contact with a SARS patient. Transmission from an unknown, but persisting animal reservoir might explain this finding; however, a nonspecific case definition (i.e., many “cases” might not actually be SARS) and limitations in contact-tracing capacity are other potential explanations.

Finding a potential animal source is, however, a daunting task. The province is famous for its “wet markets,” where a bewildering variety of live fauna are offered for sale (sometimes illegally) for their medicinal properties or culinary potential. The opportunity for contact, not only with farmed animals but also with a variety of otherwise rare or uncommon wild animals, is enormous. More than one third of early cases, with dates of onset before February 1, 2003, were in food handlers (persons who handle, kill, and sell food animals, or those who prepare and serve food) (Guangdong Province Center for Disease Control and Prevention, unpub. data,).

Hypothesis-generating epidemiologic studies should be carried out immediately in China, focusing on early cases of SARS and cases in persons without known contact with infected persons. These studies should also collect information from appropriately selected controls (i.e., matched by categories such as community and age), regarding exposures to animals of any kind in any setting (including food preparation, dietary habits, pets, and a variety of other activities and behaviors in the community).

Plausible hypotheses generated by epidemiologic studies should be briskly followed by intensive, focused, laboratory studies where relevant, including surveys of specific animal populations to identify SARS-associated coronaviruses (by culture and polymerase chain reaction [PCR]) or to measure specific antibodies. Some virologic surveys have already been conducted among prevalent animal populations, including those known to harbor other coronaviruses or other viruses transmissible to humans or wild animals, handled and sold in the markets; a variety of animals have been reported to harbor SARS-associated coronavirus. However, whether these animals are transmitting virus or are recipients of virus transmission is not yet clear. Solutions will lie with identifying epidemiologic links, which should guide targeted animal studies. Molecular epidemiologic and genetic studies can then be helpful in evaluating viruses isolated from animals and from humans.

## Natural History of the Epidemic

Since the earliest known cases were in Guangdong Province, China has had more time than any other location to observe disease incidence over time. Evidence from Guangdong Provincial Centers for Disease Control suggests that the disease incidence peaked in mid-February, and declined weekly through May. What were the reasons for the decline? Introduction of stringent infection-control measures in hospital settings undoubtedly resulted in reduced incidence in healthcare settings but would not likely have accounted for reductions in community transmission. Efforts have been made to reduce the interval between onset of illness and hospitalization (minimizing the potential for community transmission). This effort likely had substantial impact in reducing disease incidence, as shown elsewhere (*6*).

The initial hypothesis was that the virus attenuated after multiple generations of transmission; this hypothesis now seems unlikely. We note several other considerations. Were there a limited number of susceptible people within the population to begin with? Such a concept is possible if there had been earlier spread of a less virulent coronavirus, providing some immunity to a proportion of the population. If so, whether this occurrence was unique to Guangdong will be important to determine.

Alternatively, did the population develop widespread immunity to the causative agent itself? This scenario would require a good deal of asymptomatic or mildly symptomatic disease. At this stage, no reason exists to exclude the possibility of a much wider spectrum of disease than is currently appreciated, since the spectrum of illness has not been fully evaluated.

Another possibility is that a second agent might be required, in addition to coronavirus, to produce severe illness; if this is the case, the epidemiology (like seasonality) of the second agent (perhaps a less recently emerged pathogen for which there is already fairly widespread immunity), rather than coronavirus, may actually be responsible for the decline of the incidence of SARS in Guangdong.

Extensive seroprevalence studies will be helpful for sorting through these possibilities. Analyzing stored serum samples, collected before the onset of this outbreak, could be of immense value in evaluating the possibility of preexisting immunity. Some researchers have found human metapneumoviruses (*7*) and species of *Chlamydia* in patients with SARS, but the importance of these findings is unclear. Systematic evaluation of specimens available from all cases, severe cases, and healthy controls in China regarding the presence of antibodies to coronavirus, as well as hypothesized co-infecting agents, should be done. Important clues may come from seroprevalence and other epidemiologic studies in children. As in other affected countries, children were disproportionately less affected by SARS than adults. Carefully working through the bases for reduced incidence and severity may uncover cross-protecting infectious or immunizing agents or crucial host factors for protection.

## “Super-Spreaders”

When documenting the source of person-to-person transmission of SARS has been possible, a substantial proportion of cases have emanated from single persons, so-called super-spreaders (*l*). While contact tracing is undoubtedly incomplete, most infected patients have transmitted illness to few other people. Understanding the differentiating characteristics of persons who transmit, especially patients who are able to transmit to several other people, often after minimal contact, may provide important clues for public health strategies focused on preventing transmission. In addition, better defining environmental settings or circumstances that facilitate high transmission rates would be helpful. China is not unique in documenting super-spreaders. The country could participate in multinational studies to define the characteristics of super-spreaders and their role in the epidemiology of SARS. Of particular interest is the virus load of super-spreaders, compared with those of other infected persons.

Little is known about the importance of fecal-oral transmission or about the length of time that virus shedding occurs in the gastrointestinal tract. Virus shedding in feces has major implications for control strategies and for the possibility of continued carriage and shedding by clinically recovered patients. China has the opportunity to explore the role of fecal spread in the transmission of SARS.

## Evolution of the Virus

The causative agent is a coronavirus (*8*–*10*), and the entire genome of several strains has been fully sequenced by many laboratories globally (*2*,*3*,*11*). Tests have been developed to detect coronavirus genetic sequences by PCR. In addition, tests to detect SARS-associated coronavirus antibodies have been developed, but the sensitivity and specificity of these tests are low, especially early in the illness when public health and clinical needs are greatest. A good test for SARS would be important not only for diagnosis and management but also for investigating the origin of the disease and for defining its epidemiology.

If the causative agent can be isolated from stored specimens from the earliest group of patients (from November 2002 to January 2003), how their genetic sequences compare with those from viruses isolated later from various parts of China and elsewhere, and from animals from Guangdong and Guanxi Provinces, would be useful to know. Mutations may be important for a number of reasons. They may affect transmissibility and virulence; they may provide (or frustrate) therapeutic targets for new drugs; and they may pose challenges for development of diagnostic tests and vaccines. Specimens from Chinese patients provide the longest observation window with which mutational tendencies can be evaluated.

An analysis of 14 full-length sequences suggests that two genetic lineages might have arisen from Guangdong. One lineage is represented by the chain of transmission associated with the physician from Guangzhou who traveled to Hong Kong, Special Administrative Region, in February. The other lineage is associated with isolates from Hong Kong, Guangzhou, and Beijing (*11*). If two genetic lineages arose in Guangdong, were there two separate transmission events from an animal host to humans, or did the lineage diverge within humans? Specimens from early cases in Guangdong may be helpful in addressing this question.

## Outcomes of Infection

Epidemiologic, immunologic, and microbiologic factors associated with severe outcome are not fully defined. Clearly, though, a principal determinant for poor outcome is advancing age. As with other respiratory diseases, age-related coexisting conditions reduce the capacity to compensate to conditions associated with severe disease. Understanding other specific factors that result in poor outcome will have value for optimizing therapeutic approaches.

Experienced clinicians disagree about the value of early treatment with ribavirin and high-dose corticosteroids, and some are reticent to ventilate patients because of high risk for transmission to healthcare workers associated with intubation. More data are needed to help define the most effective treatment strategy, particularly for areas with limited resources.

Extraordinary clinical expertise exists among health professionals in Guangdong Province. They have substantial experience with a variety of antivirals, antibiotics, alternative (herbal) medicines, and corticosteroids, and with using assisted ventilation in the treatment of patients with SARS (*12*). While randomized clinical trials have not been conducted, careful compilations of existing case series data would be helpful in evaluating the potential effectiveness of various management regimens.

The store of clinical data, accumulated from treating hundreds of SARS cases, needs to be put to good use. One priority is to investigate clinical, epidemiologic, and laboratory predictors of poor outcome. Such experience will supplement other recently published data from Hong Kong, Special Administrative Region (*1*,*13*–*15*), and Singapore (*16*).

Several questions remain unanswered. Do patients exposed to high viral doses (for which a short incubation period may be a surrogate) or to a co-infecting pathogen have poorer outcomes? What is the impact of multiple exposures to SARS-associated coronavirus, like that which occurred among healthcare workers early in the epidemic? Do patients infected early in the transmission cycle perform more poorly than those infected during subsequent cycles of transmission?

## Learning from the SARS Epidemic

Seldom have intersections between politics, economic development, and public health been more graphically demonstrated. While awaiting the development of effective prophylactic and therapeutic options, many countries have had to muster substantial political will for quick and transparent steps to declare the presence of a lethal pathogen within their borders; conduct surveillance and report the results; use contact tracing, quarantine, and border control measures when needed; and apply stringent infection control measures in healthcare settings. Providing the general public with timely and candid information about the magnitude of the problem, the known risks, and how persons can protect themselves has also been necessary. These actions were necessary even when they appeared contrary to economic interests in the short run. As has been shown in China, delaying implementation can result in disastrous public health consequences extending beyond its borders, in addition to damage to the economy and national image.

The work outlined here involves descriptive and epidemiologic inquiry, fundamental to establishing an understanding of this new pathogen and disease. While refined and esoteric research will likely also be conducted, support must first be established for systematically addressing these basic questions and rapidly disseminating results through publication in international journals, presentations at international meetings, and in public communications. In China, in contrast with many other settings globally, scientific inquiry and dissemination of results to the international community are subject to institutional interference. The SARS pandemic has shown that virulent pathogens are beholden to no political philosophy or edict. Only careful and rapid application of knowledge and reason through a variety of public health measures has been effective in minimizing the spread and severity of the SARS epidemic. More information and data generated from studies of the epidemic in China are needed immediately to save lives and to prevent fear and disease, both in China itself and elsewhere in the world.

SARS became a public health emergency for China, where investment in health services has been given low priority for many years. Maintaining control in a country so large and diverse will be a major challenge for the months, and perhaps years, to come. Each of China’s mainland provinces (including municipalities with equivalent status, autonomous regions, and special administrative regions) is like a country within a country. Many are larger than most countries in Europe. Some, such as Shanghai, are wealthy and highly developed, while others such as Guangxi (bordering Guangdong and Vietnam) are poor and typical of developing countries. Given the potential for reemergence of SARS in the future, if sustained control measures are not in place in China, the possibility of controlling the global threat posed by the disease until new technology (i.e., an effective vaccine) is available may be slight. Key strategies include effective disease surveillance and reporting with early detection and isolation; hospital infection control during triage and treatment of cases; and transparent, open public communication about risk and disease magnitude.

China has recently begun to vigorously address the need for better surveillance, accurate reporting, and forthright public communication. Substantial epidemiologic, clinical, virologic, and immunologic expertise and interest are available within China to address the fundamental questions. International expertise is also available to provide guidance, feedback, and assistance when requested. Identifying the modest resources needed to implement the work should not be a barrier. Support from the government will be needed to carry out valid, transparent studies, and for permission to report the findings, regardless of the conclusions. SARS provides a jarring reminder of the preparedness that is needed to respond to emerging and existing disease threats; it highlights the need to reinvest in health in China, and strengthen public health programs, including surveillance systems and response capacity.

While disease incidence has abated in China and in other locations globally, the disease may still represent an important threat in the future. Many of the solutions to solve the multifaceted puzzle of SARS and to prevent future epidemics must come from China. Without solutions from that country, the degree of difficulty for sustained control of the problem globally is raised still higher.

## References

[1] Tsang KW, Ho PL, Ooi GC, Yee WK, Wang T, Chan-Yeung M, A cluster of cases of severe acute respiratory syndrome in Hong Kong. N Engl J Med. 2003;348:1977–85. 10.1056/NEJMoa030666 12671062

[2] Marra MA, Jones SJ, Astell CR, Holt RA, Brooks-Wilson A, Butterfield YS, The genome sequence of the SARS-associated coronavirus. Science. 2003;300:1399–404. 10.1126/science.1085953 12730501

[3] Rota PA, Oberste MS, Monroe SS, Nix WA, Campagnoli R, Icenogle JP, Characterization of a novel coronavirus associated with severe acute respiratory syndrome. Science. 2003;300:1394–9. 10.1126/science.1085952 12730500

[4] Ludwig B, Kraus FB, Allwinn R, Doerr HW, Preiser W. Viral zoonoses—a threat under control? Intervirology. 2003;46:71–8. 10.1159/000069749 12684545

[5] Williams ES, Yuill T, Artois M, Fischer J, Haigh SA. Emerging infectious diseases in wildlife. Rev Sci Tech. 2002;21:139–57. 11974625 10.20506/rst.21.1.1327

[6] Riley S, Fraser C, Donnelly CA, Ghani AC, Abu-Raddad LJ, Hedley AJ, Transmission dynamics of the etiological agent of SARS in Hong Kong: impact of public health interventions. Science. 2003;300:1961–6. 10.1126/science.1086478 12766206

[7] Poutanen SM, Low DE, Henry B, Finkelstein S, Rose D, Green K, Identification of severe acute respiratory syndrome in Canada. N Engl J Med. 2003;348:1995–2005. 10.1056/NEJMoa030634 12671061

[8] Peiris JS, Lai ST, Poon LL, Guan Y, Yam LY, Lim W, Coronavirus as a possible cause of severe acute respiratory syndrome. Lancet. 2003;361:1319–25. 10.1016/S0140-6736(03)13077-2 12711465 PMC7112372

[9] Ksiazek TG, Erdman D, Goldsmith CS, Zaki SR, Peret T, Emery S, A novel coronavirus associated with severe acute respiratory syndrome. N Engl J Med. 2003;348:1953–66. 10.1056/NEJMoa030781 12690092

[10] Drosten C, Gunther S, Preiser W, van der Werf S, Brodt JR, Becker S, Identification of a novel coronavirus in patients with severe acute respiratory syndrome. N Engl J Med. 2003;348:1967–76. 10.1056/NEJMoa030747 12690091

[11] Ruan YJ, Wei CL, Ee LA, Vega VB, Thoreau H, Su ST, Comparative full-length sequence analysis of 14 SARS coronavirus isolates and common mutations associated with putative origins of infection. Lancet. 2003;361:1779–85. 10.1016/S0140-6736(03)13414-9 12781537 PMC7140172

[12] Zhong NS, Zeng GQ. Our strategies for fighting severe acute respiratory syndrome (SARS). Am J Respir Crit Care Med. 2003;168:7–9. 10.1164/rccm.200305-707OE 12773318

[13] Lee N, Hui D, Wu A, Chan P, Cameron P, Joynt GM, A major outbreak of severe acute respiratory syndrome in Hong Kong. N Engl J Med. 2003;348:1986–94. 10.1056/NEJMoa030685 12682352

[14] Donnelly CA, Ghani AC, Leung GM, Hedley AJ, Fraser C, Riley S, Epidemiological determinants of spread of causal agent of severe acute respiratory syndrome in Hong Kong. Lancet. 2003;361:1761–6. 10.1016/S0140-6736(03)13410-1 12781533 PMC7112380

[15] Peiris JSM, Chu CM, Cheng VCC, Chan KS, Hung IFN, Poon LLM, Clinical progression and viral load in a community outbreak of coronavirus-associated SARS pneumonia: a prospective study. Lancet. 2003;361:1767–72. 10.1016/S0140-6736(03)13412-5 12781535 PMC7112410

[16] Hsu LY, Lee CC, Green JA, Ang B, Paton NI, Lee L, Severe acute respiratory syndrome (SARS) in Singapore: clinical features of index patient and initial contacts. Emerg Infect Dis. 2003;9:713–7. 12781012 10.3201/eid0906.030264 PMC3000162

